# Evaluation of score parameters for severity assessment of surgery and liver cirrhosis in rats

**DOI:** 10.1017/awf.2023.21

**Published:** 2023-03-10

**Authors:** Johanne C Krueger, Moriz A Habigt, Marius J Helmedag, Moritz Uhlig, Michaela Moss, André Bleich, René H Tolba, Rolf Rossaint, Marc Hein, Mare Mechelinck

**Affiliations:** 1 Institute for Laboratory Animal Science and Experimental Surgery, RWTH Aachen University, Faculty of Medicine, Aachen 52074, Germany; 2 Department of Anaesthesiology, RWTH Aachen University, Faculty of Medicine, Pauwelsstraße 30, Aachen 52074, Germany; 3 Department of General, Visceral and Transplantation Surgery, RWTH Aachen University, Faculty of Medicine, Aachen 52074, Germany; 4 Institute for Laboratory Animal Science and Central Animal Facility, Hannover Medical School, Hannover 30625, Germany; 5 Animal Welfare Unit, University of Bonn, Bonn 53113, Germany

**Keywords:** animal welfare, human endpoints, liver cirrhosis, rats, score-sheet, severity assessment

## Abstract

Severity assessment in animals is an ongoing field of research. In particular, the question of objectifiable and meaningful parameters of score-sheets, as well as their best combination, arise. This retrospective analysis investigates the suitability of a score-sheet for assessing severity and seeks to optimise it for predicting survival in 89 male Sprague Dawley rats (*Rattus norvegicus*), during an experiment evaluating the influence of liver cirrhosis by bile duct ligation (BDL) on vascular healing. The following five parameters were compared for their predictive power: (i) overall score; (ii) relative weight loss; (iii) general condition score; (iv) spontaneous behaviour score; and (v) the observer’s assessment whether pain might be present. Suitable cut-off values of these individual parameters and the combination of multiple parameters were investigated. A total of ten rats (11.2%; 10/89) died or had to be sacrificed at an early stage due to pre-defined humane endpoints. Neither the overall score nor any individual parameter yielded satisfactory results for predicting survival. Using retrospectively calculated cut-off values and combining the overall score with the observer’s assessment of whether the animal required analgesia (dipyrone) for pain relief resulted in an improved prediction of survival on the second post-operative day. This study demonstrates that combining score parameters was more suitable than using single ones and that experienced human judgement of animals can be useful in addition to objective parameters in the assessment of severity. By optimising the score-sheet and better understanding the burden of the model on rats, this study contributes to animal welfare.

## Introduction

The issue of severity experienced by animals has been widely discussed (Bleich & Tolba 2017). The EU Directive 2010/63 mandates both upfront and retrospective assessment of the severity to which an animal is subjected during different procedures of animal experiments and provides the severity levels: ‘non-recovery’, ‘mild’, ‘moderate’ and ‘severe’ (Lindl & Gross *et al*. 2012; Maisack 2015). The Directive does not specify how the severity must be assessed. Nevertheless, it is difficult to correctly detect, determine and evaluate the different degrees of severity experienced by an individual animal, which include pain, suffering, distress and lasting harm.

Score-sheets are a commonly used tool to monitor observations regarding the severity for animals during experiments by assigning scores to pre-defined parameters and combining these scores in the end. In addition, humane endpoints are defined to avoid unnecessary suffering of animals (refinement strategy according to the 3Rs principle [refinement, replacement and reduction]) (Russell & Burch 1959). As far back as 1985 Morton and Griffiths suggested a hypothesis for researchers to determine and implement parameters specific to the experiments conducted. In that publication, they also presented a concrete exemplary score for the overall animal assessment with different parameters. Nevertheless, to date, these parameters have not been reasonably validated, neither individually nor in combination with each other. Moreover, answering many research questions requires a combination of several surgical interventions, multiple anaesthesia or the performance of an intervention in the presence of a pre-induced underlying disease (e.g. cirrhosis of the liver), which affects the severity of the procedures and further complicates a correct assessment.

The aim of this study was to retrospectively investigate, in an experiment with two consecutive surgeries: (i) sham surgery or bile duct ligation (BDL); and (ii) balloon dilatation of the carotid artery, the suitability of a semi-quantitative score-sheet with regards to the used parameters and avoid unnecessary euthanasia by optimising cut-off values and combining parameters. The aim of retrospectively identifying cut-off values as predictive red flags for expected animal death is to provide researchers of future similar studies with reference data when deciding whether to kill an experimental animal. This is to prevent expected animal suffering. Secondary objectives were to investigate the effect of surgery type, induced pathologic state (liver cirrhosis) and second hit (second surgery) on score parameters and mortality.

We hypothesised that by combining individual parameters and adding additional parameters, the semi-quantitative score-sheet could be retrospectively improved to better predict death as an outcome and avoid misleading judgements.

## Materials and methods

### Study animals

All experiments were approved by the governmental animal care and use committee (No 84-02.04.2016.A391 Landesamt für Natur, Umwelt und Verbraucherschutz Nordrhein-Westfalen, Germany). The German Animal Welfare Act, the EU Directive 2010/63, as well as the *Guide for the Care and Use of Laboratory Animals* were followed in all study protocols. The study protocol has not been published or registered in advance. This manuscript adheres to the ARRIVE guidelines (Percie du Sert 2020).

The purpose of the reported animal experiments was to evaluate the effects of liver cirrhosis on vascular remodelling in male Sprague Dawley rats (*Rattus norvegicus*; RjHan:SD; Janvier Labs, Le Genest-Saint-Isle, France). These results will be published separately. During the study we got the impression that the actual score-sheet was not ideal to display severity and predict mortality. According to the 3Rs principle and to help improve animal welfare during experiments, the rat scoring and outcome data were additionally used for this retrospective study.

**Figure 1.**
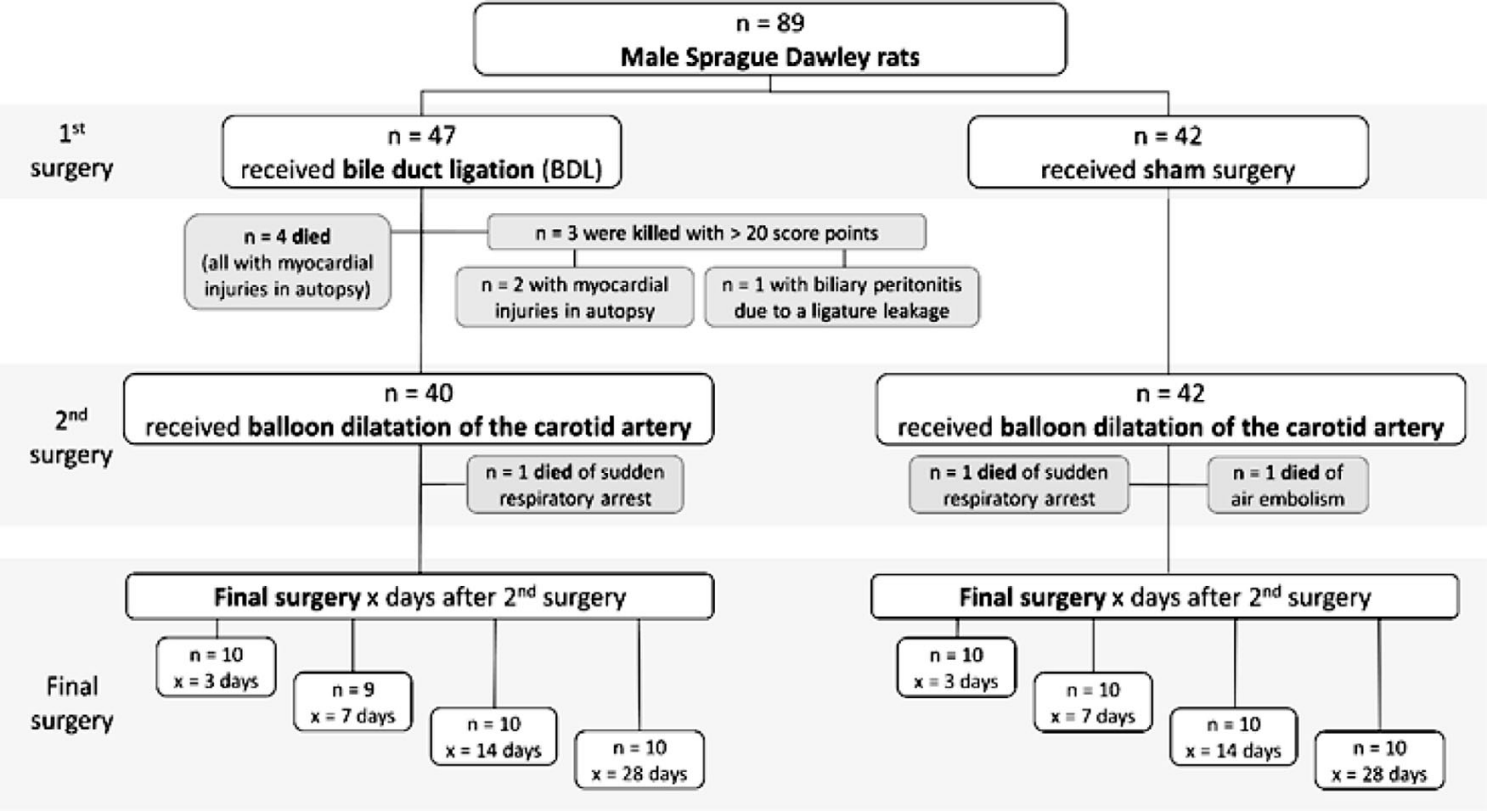
Flow chart of the experimental design showing the number (n) of rats for the two groups (sham and BDL) within the 1st and 2nd surgery, and the experimental duration after which pre-defined euthanasia was performed. In addition, causes and number of deaths or early euthanasia are presented shaded in grey

Following at least seven days of acclimation, the rats were quasi-randomly assigned to undergo bile duct ligation (BDL) or sham surgery. Blinding was not possible as the rats were visually distinguishable after the procedure. The entire study cohort comprised 89 animals, including 47 rats subjected to BDL and 42 to sham surgery (Figure 1). To compensate for premature death of a rat and avoid compromising statistical power, the authorities pre-approved a total of 16 substitutes for this experiment (eight per group). Therefore, the difference in group size resulted from premature death or euthanasia of rats (Figure 1) and consequently the use of substitute animals.

Each animal underwent two different procedures during the entire experiment: either BDL to induce liver cirrhosis or sham intervention, and for all animals, balloon dilatation of the left carotid artery four weeks after the first surgery (Abarbanell 2010; Holt & Tulis 2013; Yang *et al*. 2014; Zhang & Trebak 2014). The contralateral (right) carotid artery could be used as a control for the effect of vascular damage, therefore, sham surgery for balloon dilatation was not reasonable in the sense of the 3Rs principle (Russell & Burch 1959). Each group (BDL and sham) was divided into four subgroups depending on the experimental duration (Figure 1). The experimental duration was defined as the period from the second surgery to euthanasia and was 3, 7, 14 or 28 days after the second surgery. The animals were randomised into these sub-groups after the second surgery to minimise performance bias.

Confounding factors were minimised by standardising the treatment of animals in terms of food, water, cage cleaning, cage size, environment, lighting, temperature, transport methods, timing of procedures, and surgical procedures. All animals were housed in groups of two or three under specific pathogen-free conditions in filter-top cages (Type 2000 cages; Tecniplast, Hohenpeissenberg, Germany) that were sanitised twice a week. In the immediate post-operative phase, animals were separated for up to three days until full recovery. Environmental temperature and humidity were kept constant (22°C; range 20–24°C and 55%; range 45–65%, respectively), and a 12/12h light/dark cycle was employed. Sterile, acidified water and standard pellets for laboratory rats (Ssniff GmbH, Soest, Germany) were provided *ad libitum.*

### Anaesthesia and monitoring

Anaesthesia was performed identically for all interventions. For intra-operative analgesia, buprenorphine was injected subcutaneously prior to surgery (ESSEX PHARMA, Munich, Germany; 0.01 mg kg^–1^ bodyweight [BW]). After 30 min, rats were sedated in a specialised box for anaesthetic induction using an oxygen flow rate of 4 L min^–1^ and 4 vol% isoflurane (Forene®, 100% v/v, AbbVie, Wiesbaden, Germany). Anaesthesia was maintained using an oxygen flow rate of 2 L min^–1^ and 2–3 vol% isoflurane via a nose cone (HSE Anaesthesia Mask, Harvard Apparatus GmbH, Hugstetten, Germany). Rats breathed spontaneously at all times. Dipyrone (subcutaneous, 100 mg kg^–1^ BW, diluted to 100 mg mL^–1^; Novaminsulfon-ratiopharm® 1 g 2 mL^–1^, Ratiopharm, Ulm, Germany) was injected 30 min before the end of anaesthesia as supplementary post-operative analgesia. Additionally, a local infiltration of ropivacaine 0.5% (25 mg kg^–1^ BW; Ropivacain Kabi 10 mg mL^–1^, Fresenius Kabi AG, Kriens, Switzerland) was administered. Eye ointment (Bepanthen®, Bayer, Leverkusen, Germany) was used after anaesthetic induction to avoid corneal damage. Adequate depth of anaesthesia was confirmed by the absence of any nociceptive response to tail-tip or inter-digital pinching. To monitor the animals during anaesthesia, electrocardiograms were recorded using needle electrodes, and peripheral oxygen saturation was measured using pulse oximetry at the paw (Masimo Radical 7 Blue Screen, Irvine, USA) as described previously (Mechelinck 2019). Body temperature during surgeries was controlled using a rectal temperature probe connected to a feedback-controlled heating pad (TCAT-2LV controller, Physitemp, Clifton, USA). After surgery, rats were allowed to recover in a small animal intensive care unit with warm air (around 30°C) and elevated oxygen levels (oxygen flow rate was adjusted to 1 L min^–1^ which provided an oxygen level of approximately 40–45%) until full recovery (Vetario, Weston-super-Mare, UK).

### Protocol and procedures

All procedures were performed under general anaesthesia. All interventions started between 0800 and 1400h.

For BDL, after anaesthesia induction, the abdomen was disinfected, and median laparotomy was performed. The bile duct was identified, exposed, ligated twice with 5/0 silk suture (18020-50, Fine Science Tools, Vancouver, Canada) and sectioned between the two ligatures. The cranial of the two ligations was placed directly after the confluence of the bile ducts to minimise cyst formation in the common bile duct and thus increase the survival rate (Yang *et al*. 2015). Ligature and trans-sectioning were omitted in the sham group.

To prevent haemorrhage caused by reduced synthetic capacity of the liver, all animals were subcutaneously administered vitamin K (2.5 mg kg^–1^ BW; PZN 04273031, phytomenadione [Konakion®] MM 10 mg mL^–1^, F Hoffmann-La Roche AG, Basel, Switzerland) weekly throughout the study period (Akimoto *et al*. 2005).

Balloon dilatation of the left carotid artery (second surgery) was performed using the same anaesthesia protocol described earlier. The carotid artery was accessed through a median incision in the ventral neck followed by a blunt preparation. For sampling 1.9 mL of blood and volume replacement (Sterofundin® ISO 1/1 E ISO, B Braun Melsungen AG, Melsungen, Germany), a central venous catheter (CVC) (1 Lumen catheter set, Leaderflex 22G, VYGON GmbH & Co KG, Écouen, France) was placed in the left external jugular vein. A 2-French Fogarty catheter was inserted into the left external carotid artery (ECA), advanced up to the aorta, inflated to a pressure of 2.0 atm and retracted three times with a rotational movement up to the entry point of the catheter. At the end of the procedure the ECA was ligated. Common carotid artery perfusion was restored via the internal carotid artery. The skin wound was repaired in two layers.

After the pre-defined survival time according to the four different groups (experimental duration of 3, 7, 14, 28 days), the animals were given a final anaesthetic. Blood samples were taken, and the rats were then killed via exsanguination through removal of the heart under deep anaesthesia. Liver tissue samples were collected for histological examinations and weighed.

### Follow-up care

Post-operatively, all animals were examined after 6 to 8 h and were subsequently weighed and scored at least once daily (in the morning). For overall scores of ≥ 10, assessments were performed at least twice daily (in the morning and evening).

For animal scoring, a semi-quantitative, multi-modal score-sheet (Table 1) was used. It was adapted from former score-sheets inspired by suggestions (Morton & Griffiths 1985) and adjusted to model-specific needs. It comprised four categories (BW, general state, spontaneous behaviour and procedure-specific parameters), each with different parameters. Numeric score values were assigned to these parameters. The footnotes in Table 1 were added retrospectively as part of this study to clarify the authors’ understanding of certain terms. For each animal, all individual score values were summed up daily to obtain an overall score. Depending on the value of this overall score, pre-defined measures listed in section 5 of Table 1 were carried out. For calculating the score points for BW, BW was compared daily with the highest previously measured BW to calculate the percentage BW loss. The score sheet allowed > 20% BW loss by the second post-operative day compared to the condition before surgery. A score of 19 was assigned to this BW loss to avoid exclusion of animals losing BW solely because of an intervention (including anaesthesia). The scores of animals additionally exhibiting other signs of severity were then allowed to reach the pre-defined humane endpoints (20 points). In general, scores exceeding 20 were defined as the humane endpoint, and animals were euthanased immediately.

**Table 1.** Score-sheet.

Parameter	Score
I. Bodyweight	
– Unaffected or increase	0
– < 10% weight loss	5
– 11–20% weight loss	10
– >20% weight loss for no more than two post-operative days	19
II. General condition	
– Fur smooth and shiny, body orifices clean	0
– Fur ruffled, lustreless, eyes dull	3
– Moist and sticky body orifices (including chromodacryorrhoea)	5
– Abnormal posture^1^, increased muscle tone	7
– Dehydration (standing cutaneous fold)	10
– Cramps, paralysis, respiratory sounds^2^, cold temperature^3^	20
III. Spontaneous behaviour	
– Normal behaviour (sleep, reaction to blow and touch, curiosity, social contact)	0
– Abnormal behaviour^4^	2
– Impaired motor function^5^ or hyperkinesia	5
– Isolation, pain noises^6^	6
– Apathy, distinct hyperkinetic, stereotypy^7^, co-ordination disorder	10
Automutilation	20
IV. Procedure-specific parameters	
– Bleeding from mouth, nose, anus: slight (no bleeding after removal)	10
– Bleeding from mouth, nose, anus: severe (continuous blood loss)	20
– Signs of a severe stroke^8^	20
– Tense abdominal wall	10
∘ With limited ventilation (breathing through the open mouth, peripheral oxygen saturation < 90%)	20
– Signs of encephalopathy (these are included under III, hence no score points here)	
V. Assessment and actions	
– No severity	0–4
– Mild severity: thorough the observation of the animal (at least once a day), if necessary supporting actions (e.g. heat supply or special diet)	5–9
– Moderate severity: starting medical treatments (e.g. analgesia, antibiosis or fluid therapy) if necessary, increasing the observation intervals to at least twice daily; categorised as severe strain if this state lasts for > 72 h	10–19
–Severe severity: animal must be euthanased	>20

1 This includes for example back arching, tense abdominal wall and lack of muscle tone.

2 Such as wheezing, gasping for air or grunting on expiration, often associated with accelerated breathing.

3 Detection of severe deviations of the normal temperature was performed by touching the animals while handling for weighing.

4 This includes, for example, less rearing, decreased activity and a reluctance to move, lack of curiosity, lack of drinking or eating, eating of bedding material, lack of reaction to touch or blow, excessive sleeping, social distancing, writhing, or increased aggression.

5 Such as gait disorders (including lameness), clumsiness, balance problems and unsteady movements.

6 ‘Pain noises’ refers to audible squeaks, which can be a nociceptive response.

7 This refers to repetitive and unchanging actions (such as continuous sniffing, licking, biting, compulsive gnawing or other compulsive motor movements) without goal or function.

8 Such as convulsions, inability to react, hemiparesis or tetraplegia.

Seven different researchers were involved in scoring the rats. The observations were not blinded, since animals of the different groups were visually distinguishable (by jaundice of the BDL rats). To minimise inter-observer variability, visits and assessments were performed as a team at the beginning, and the procedure was agreed upon by the team. Subsequently, each scoring was carried out by one person. A second person was consulted when difficulties occurred, in unclear situations or for borderline cases.

On the day of surgery, post-operative subcutaneous dipyrone re-injection was performed once as a standard and subsequently only as needed, depending on the general state and behaviour of the animal. The decision to apply analgesics was based on the observers’ subjective assessment and was made very liberally across all examiners. Since signs of pain were included in all categories of the score-sheet, it was not possible to identify a single sign of pain. Instead, all signs of pain and discomfort shown by the animal were included in the decision-making. In the case of unusual behaviour (e.g. unspecific signs for a reduced well-being, such as reduced mobility) for no apparent reason, it was assumed that pain could not be ruled out (benefit of the doubt; Hawkins *et al*. 2011). In order to take subtle signs into account, the examiner was given the option of administering analgesics if the animal appeared conspicuous in its overall appearance, even if all the individual signs did not formally show any score points. The ‘daily analgesic requirement as assessed by the observer’ (DARAO) was used retrospectively as an additional indicator of severity.

### Sample processing and histological image analysis

A detailed description of the procedures for verifying the expected liver changes can be found in the Supplementary material (S1). The following parameters of clinical chemistry were measured: serum alkaline phosphatase (AP), aspartate aminotransferase (AST), alanine aminotransferase (ALT), gamma-glutamyl transferase (GGT), lactate dehydrogenase (LDH), albumin, total bilirubin, creatinine, lactate and urea levels.

In the histological specimens of the 28-days groups, fibrosis was quantified in a standardised manner and liver changes were evaluated by a blinded veterinary pathologist using a score developed for this purpose.

### Software

Statistical analyses were performed using SPSS 27 (IBM Corporation, Armonk, USA). Graphs were created using GraphPad Prism 9 (GraphPad Software, San Diego, USA).

### Statistical analysis

*A priori*, the required sample size was calculated to answer the primary research question of the original study (to detect the difference between the neointima area in the liver cirrhotic group and the control animals over a period of 28 days). This was based on an effect size estimate using data from two previous studies investigating the effect of insulin or type II diabetes on neointimal formation after 28 days (Guo *et al*. 2015) or 21 days (Park *et al*. 2001). Based on presented values, a bias-corrected effect size estimate and a standard error of the effect size estimate were calculated for both studies using an effect size calculator (https://www.cem.org/effect-size-calculator, Cambridge University, Cambridge, UK). The obtained values were used to determine the weighted effect size Hedge’s g (1.796) using a previously published method for weighting and pooling effect sizes from different studies (Turner & Bernard 2006). Using the weighted effect size in a two-tailed *t*-test in G*Power 3.1.9.7 (Heinrich-Heine-Universität Düsseldorf, Düsseldorf, Germany) with a type I error of 0.05, a power of 0.95 and an allocation ratio of 1, a sample size of ten rats per group and time-point was identified to be needed in order to detect a clinical significant difference between the groups.

Data are presented as means (± SD). Kaplan-Meier curves were used to demonstrate survival rates. Separately, for the period after each surgery, the survival curves were compared using Log-Rank-Test with the group as a categorical covariate. In the following, relative BW and relative BW loss are always given in % of the pre-operative BW. Changes of BW (both absolute and relative), overall score and DARAO were analysed using a generalised estimating equation (GEE) for group, number of surgeries and days after surgery. *T*-tests with adjustment for multiple comparison using Hochberg procedure were used for *post hoc* comparisons. To compare the values of the overall score, absolute BW, DARAO and scores for BW, general condition, spontaneous behaviour and procedure-specific behaviour measured on the second post-operative day after the first surgery, groups were compared using *t*-tests. Statistical significance was indicated by *P* < 0.05.

To retrospectively investigate whether the premature death of an animal could have been predicted with appropriate cut-off values, the day with the highest average score values and the lowest absolute BW was first identified. For the identified day, the overall score, its components and the DARAO were then compared regarding their predictive value using receiver operating characteristic (ROC) curve analyses, including areas under the curve (AUC), *P*-values, optimal cut-off values, sensitivity, specificity and positive (PPV) and negative predictive value (NPV). The optimal cut-off value was defined as the value with the maximum Youden Index (J):(1)

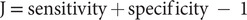

**Figure 2.**
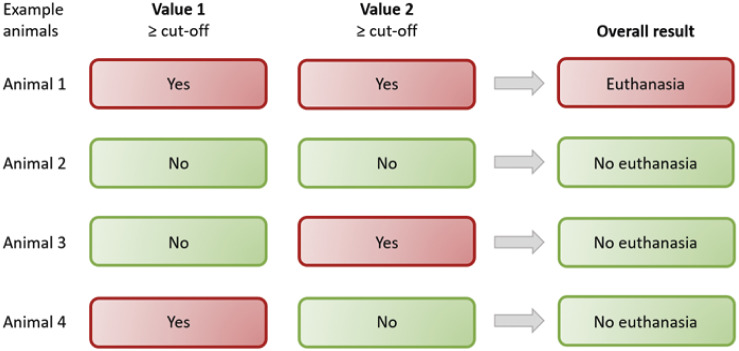
Schematic decision table for euthanasia by combining two different parameters on post-operative day 2 according to the ‘believe the negative’ rule: euthanasia is only advised if the cut-off value is exceeded for all combined parameters.

In order to reduce unnecessary euthanasia, an attempt was made to retrospectively increase the specificity of individual cut-off values by combining 2 to 3 parameters with an AUC significantly different from 0.5 (*P* ≤ 0.05). No parameters were combined that contained duplications (e.g. the overall score value with one of its components). The combination of different parameters was only considered as a decision for euthanasia in the retrospective evaluation, if all combined parameters were above the cut-off value (analogous to a ‘believe the negative’ [BTN] rule [Figure 2]) (Schink 2007; Shen 2008). For better comparability, the combinations were also evaluated for sensitivity, specificity, PPV and NPV.

**Figure 3.**
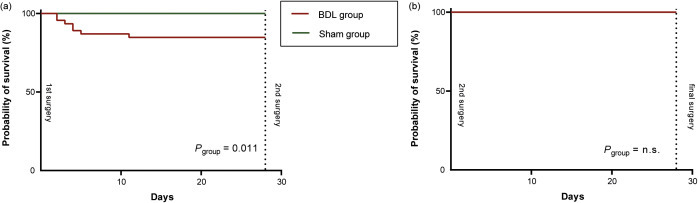
Kaplan-Meier curves of post-operative survival for the two groups (sham and bile duct ligation [BDL]) for (a) after the first surgery and (b) after the second surgery. The three animals that died due to intraoperative complications during the 2nd surgery (two sudden respiratory arrests and one air embolism) were excluded. *P*-values were calculated using Log-Rank-test, with group as covariate

## Results

### Survival

Ten (10/89; 11.24%) animals died prematurely or had to be euthanased (Figure 1): two animals (2/42; 4.76%) in the sham group and eight (8/47; 17.02%) in the BDL group. All other animals survived up to the pre-defined endpoint of the experiment (3, 7, 14 or 28 days after the second surgery). Since the three intra-operative deaths in the second surgery were most likely due to technical failure (air embolism caused by angioplasty balloon rupture) or anaesthesia associated (sudden respiratory arrest), their occurrence was neither predictable nor associated with the condition of the rats. Therefore, these three rats were excluded from further analyses in this study. Nevertheless, it is important to note that any research group should also analyse and publish intra-operative causes of death to make experiments safer and ideally avert all preventable deaths in the future. This is an important opportunity for refinement, although it is not the focus of this study.

**Figure 4.**
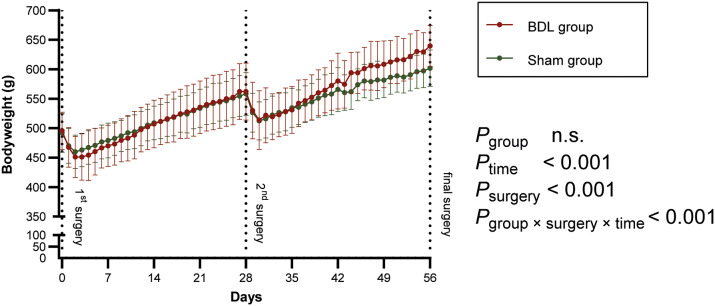
The time course of absolute bodyweight (BW) changes over the entire study period is shown for the sham (green) and bile duct ligation (BDL) groups (red). The three animals that died due to intraoperative complications during the 2^nd^ surgery were excluded. Values are presented as means (± SD). Overall *P*-values were derived from generalised estimated equations.

After the first surgery, mortality was significantly higher in the BDL group compared to the sham group (Log-Rank-Test, Chi-squared = 6.52, df = 1; *P* = 0.011) (Figure 3[a]). No rat died after the second surgery (Figure 3[b]).

### Histopathology, organ weights and clinical chemistry

BDL induced significant liver injury. The detailed results of histopathologic evaluation and analysis are shown in the Supplementary material (S2 and S3). Analysis for organ weights and clinical chemistry can be found in S4 and S5 in the Supplementary material.

### Bodyweight

At the beginning of the experiments, the mean absolute BW of all rats was 494 (± 32) g (Sham: 492 [± 33] g; BDL: 496 [± 32] g). According to the breeder (Janvier), rats of this weight are, on average, about 12 weeks old. The absolute BW did not differ significantly between the groups in the overall comparison (GEE, Wald-Chi-Square = 0.86, df *P*_group_ = 1; *P* = 0.355). After each surgery, the absolute BW first decreased and then continuously increased (Figure 4), with a significant change in absolute BW over time (GEE, Wald-Chi-Square = 3,946.37, df *P*_time_ = 27; *P* < 0.001). Absolute BW gain showed a significant difference comparing the time-period after the first to the time-period after the second surgery (GEE, Wald-Chi-Square = 89.42; df *P*_surgery*time_ = 27, *P*_surgery*time_ < 0.001). Animals of the BDL group showed a higher absolute BW over time after the second surgery (GEE, Wald-Chi-Square = 56.9; df *P*_group*surgery*time_ = 27; *P* < 0.001).

### Scores

**Figure 5.**
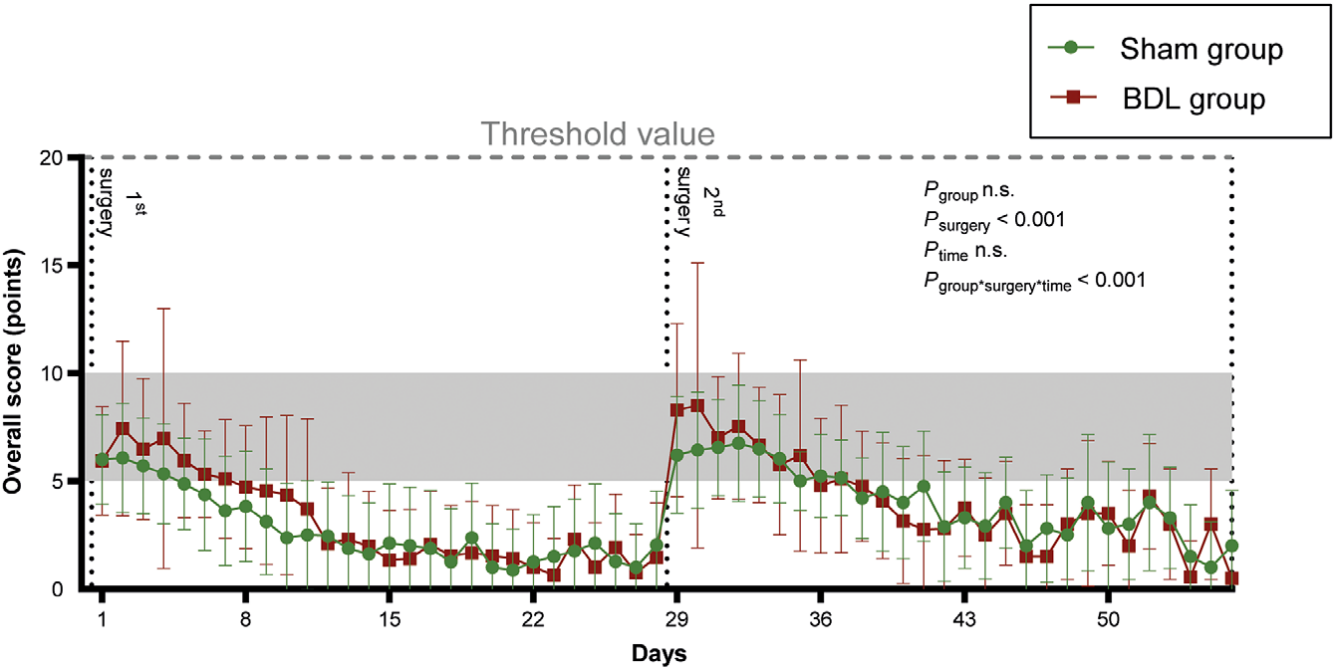
Time course of changes in the overall scores for the bile duct ligation (BDL, red) and sham groups (green). The three animals that died due to intraoperative complications during the 2nd surgery were excluded. Values are presented as means (± SD). Overall *P*-values were derived from generalised estimated equations. Mild severity is depicted by the grey-shaded area.

The overall score did not differ significantly between the two groups throughout the experiment (Figure 5) in the overall comparison (GEE, Wald-Chi-Square = 0.07, df *P*_group_ = 1; *P* = 0.789). However, the score was significantly higher after the second surgery than after the first surgery (GEE, Wald-Chi-Square = 1,071, df *P*_time_ = 27; *P* < 0.001). In the *post hoc* tests, this proved to be significant (*P* < 0.05, *t* ratio = 3.897, df = 37) only for the first post-operative day after the second surgery in the BDL group. Overall, the overall score was significantly higher after the second surgery than after the first one (GEE, Wald-Chi-Square = 25.5, df *P*_surgry_ = 1; *P* < 0.001). The interaction between group and surgery varies with respect to the overall score over time (GEE, Wald-Chi-Square = 1,635.3, df *P*_group*surgery*time_ = 27; *P* < 0.001). Additional information on the single score categories are displayed in Supplementary file 6 (S6). Notably, no animal scored points for the procedure-specific parameters in the score-sheet throughout the entire experiment. Therefore, the procedure-specific parameters were not included in individual parameter analysis.

### Daily analgesic requirement as assessed by the observer (DARAO)

Twenty-seven rats (27/86; 31.4%) (BDL: n = 20; sham: n = 7) required at least one additional analgesic treatment (subcutaneous injection of dipyrone) after one of the surgeries because of increased score levels and based on the observers’ judgement. DARAO was significantly higher in the BDL group (GEE, Wald-Chi-Square = 401.73, df = 1; *P*_group_ < 0.001) and also differed significantly on the different time-points (GEE, Wald-Chi-Square = 1,086.44, df = 4; *P*_time_ < 0.001). In the sham group, the observer assessed analgesics as required at most up to the second post-operative day, whereas in the BDL group, this continued up to the fifth post-operative day (Table 2).

**Table 2.** Number of animals that received additional dipyrone injections during the first seven days (a) after the 1st surgery (days 1–7) and (b) after the 2nd surgery (days 29–35). Dipyrone injections were given according to the observers’ assessment of analgesic requirements (DARAO). The numbers are shown separately for the bile duct ligation (BDL) and sham group

(a)						
	1 dipyrone injection		2 dipyrone injections		3 dipyrone injections	
After the 1st surgery	Sham (n)	BDL (n)	Sham (n)	BDL (n)	Sham (n)	BDL (n)
Day 1	3	5	–	–	–	1
Day 2	2	5	–	2	–	–
Day 3	–	1	–	–	–	–
Day 4	–	1	–	–	–	–
Day 5	–	1	–	–	–	–
Day 6	–	–	–	–	–	–
Day 7	–	–	–	–	–	–

(b)						
	1 dipyrone injection		2 dipyrone injections		3 dipyrone injections	
After the 2nd surgery	Sham (n)	BDL (n)	Sham (n)	BDL (n)	Sham (n)	BDL (n)
Day 29	2	7	1	3	–	–
Day 30	–	3	–	–	–	–
Day 31	–	2	–	1	–	–
Day 32	–	3	–	–	–	–
Day 33	–	1	–	–	–	–
Day 34	–	–	–	–	–	–
Day 35	–	–	–	–	–	–

The three animals that died due to intraoperative complications during the 2nd surgery were excluded. *P*-values derived from generalised estimated equations: *p*_group_ < 0.001; *p*_time_ < 0.001.

### Receiver operating characteristic (ROC) curve and cut-off analysis

**Figure 6.**
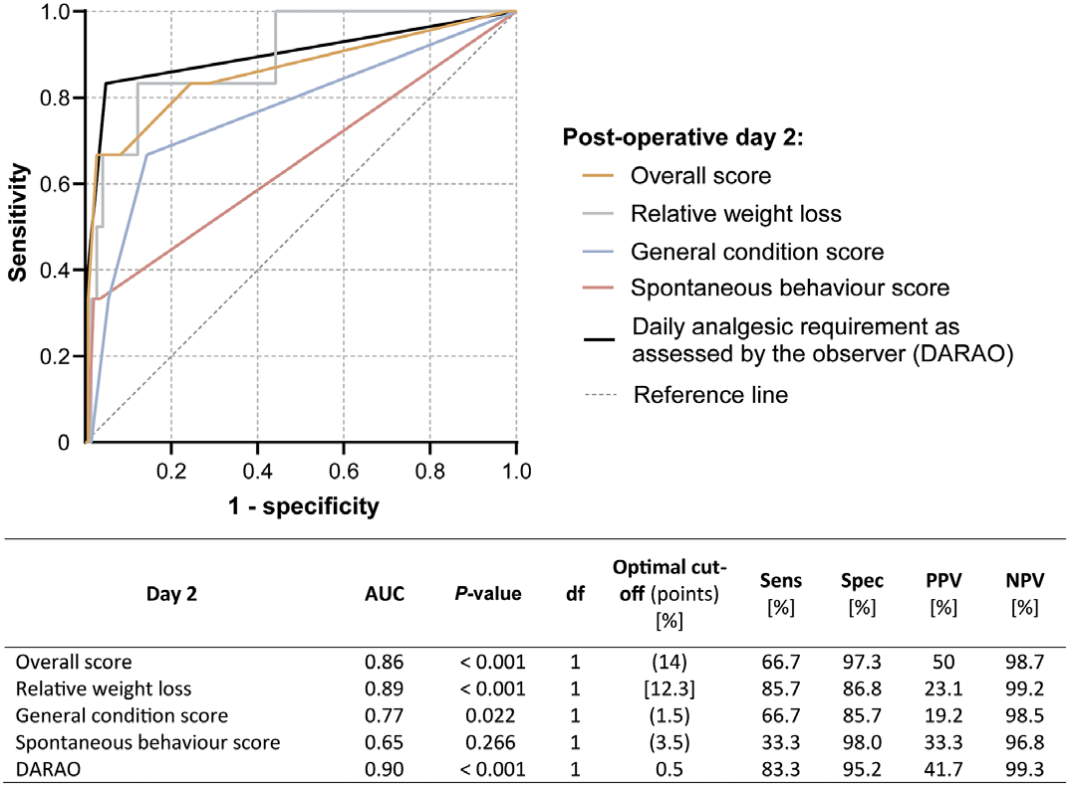
Receiver operating characteristic (ROC) curves for the overall score, separate score elements (relative weight loss, general condition score and spontaneous behaviour score), and the daily analgesic requirement as assessed by the observer (DARAO) on post-operative day 2. For each of the parameters on post-operative day 2, the table shows the area under the curve (AUC), the *P*-value, the degrees of freedom, the optimal cut-off point, sensitivity, specificity, positive (PPV) and negative predictive value (NPV).

On average, the highest score values and the lowest absolute BW were detected on the second post-operative day, and no animal died post-operatively before that day. Therefore, the absolute BW, score points and DARAO measured on the second post-operative day were contrasted (Table 3) and used to investigate whether rats, that died or had to be euthanased in the further course of the experiment, could have already been identified on that day. Results of ROC curve analyses, including AUCs, *P*-values, df, optimal cut-off values and sensitivity, specificity, PPV and NPV of the cut-off values, are shown in Figure 6. Optimal cut-offs were 14 points for the overall score, 12.3% of relative BW loss, 1.5 points for the general condition score and 0.5 DARAO per day. From the parameters investigated, the DARAO yielded the highest AUC (ROC analysis, AUC = 0.90, df = 1; *P* < 0.001), followed by relative weight loss (ROC analysis, AUC = 0.89, df = 1; *P* < 0.001) and the overall score (ROC analysis, AUC = 0.86, df = 1; *P* < 0.001). Only for the spontaneous behaviour score, the area under the curve was not significantly different from 0.5 (ROC analysis, AUC 0.65, df = 1; *P* = 0.266). The cut-off values for both the objective parameter of relative BW loss and the subjective parameter of DARAO also achieved the highest sensitivity using a single parameter (83.3% each), with a corresponding specificity of 87.8% (relative BW loss) and 95.3% (DARAO). However, the PPVs of all cut-off values of individual parameters were low and, in contrast to the other values, was highest for the overall score (PPV = 50%). The NPVs, on the other hand, were high for all parameters (at least 98.5%).

**Table 3.** The following values, measured on the second post-operative day after the first (BDL or sham) or second surgery (balloon dilatation of the carotid artery), are given for both study groups (BDL or sham): absolute bodyweight, daily analgesic requirement as assessed by the observer and score points for bodyweight, general condition, spontaneous behaviour and overall score. The three animals that died due to intraoperative complications during the 2nd surgery were excluded

	1st surgery						2nd surgery	
	Sham	BDL					Sham	BDL
		dead	survived	df	*P*-value	Cohen’s *d*		
	(n = 40)	(n = 7)	(n = 39)				(n = 40)	(n = 39)
BW absolute (g)	460 (± 28)	429 (± 18)	455 (± 36)	44	0.07	33.9	512 (± 32)	514 (± 50)
BW (score points)	5.6 (± 2.0)	9.2 (± 1.9)	5.8 (± 1.8)	44	< 0.001	1.84	6.0 (± 2.3)	6.8 (± 2.7)
GC (score points)	0.5 (± 1.5)	2.7 (± 2.1)	0.5 (± 1.5)	44	0.031	1.56	0.4 (± 1.1)	1.2 (± 3.5)
Sp-B (score points)	0 (± 0)	1.7 (± 2.4)	0 (± 0)	44	< 0.001	0.89	0.2 (± 0.9)	0.7 (± 2.4)
PS (score points)	0 (± 0)	0 (± 0)	0 (± 0)	–	–	–	0 (± 0)	0 (± 0)
Overall score (points)	6.1 ± (2.5)	13.7 (± 4.7)	6.3 (± 2.7)	44	< 0.001	3.08	6.4 (± 2.7)	8.5 (± 6.6)
DARAO (n)	0.1 (± 0.2)	1.2 (± 0.8)	0.1 (± 0.2)	43	0.015	0.33	0 (± 0)	0.1 (± 0.3)

Values are presented as means (± SD). *P*-values of the comparison (*t*-test) between values of animals that died prematurely or that were euthanased (grouped together under the heading ‘dead’) and those that survived, are given. BW: bodyweight; DARAO: daily analgesic requirement as assessed by the observer; GC: general condition; Sp-B: spontaneous behaviour; PS: procedure specific, df: degrees of freedom.

**Figure 7.**
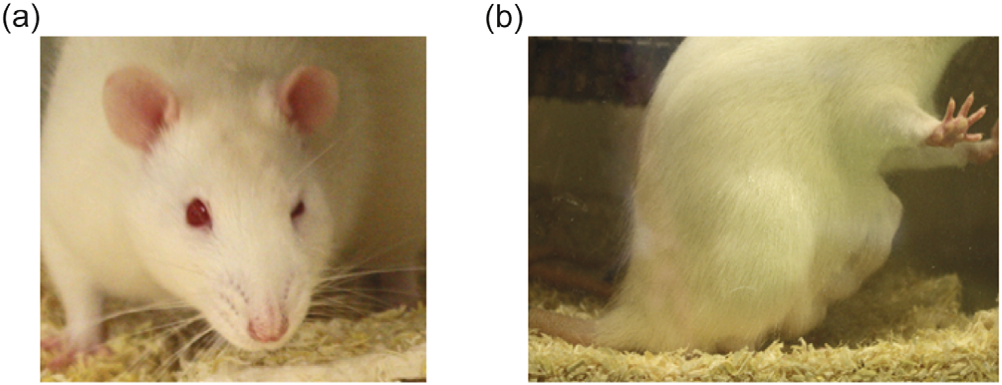
Representative cases of post-operative complications showing (a) Horner’s syndrome and (b) abdominal peritoneal rupture with herniation.

### Combining cut-off values

The combination of several parameters led to an increase in specificity, albeit at the expense of sensitivity (Table 4). A combination of the overall score and the DARAO had, in addition to a high specificity, the highest PPV (specificity and PPV each 100%), with a remaining sensitivity of 66.7% and an NPV of 98.7%. In Chi-square tests all analysed combinations proved suitable to predict mortality (Chi-square test, df = 1, Pearson’s Chi-square ≥ 39.101; all *P* < 0.001). Again, the combination of overall score and the DARAO proved to be the most suitable, based on the highest effect size (Phi = 0.811).

**Table 4.** Efficiency measures and predictive values of survival (positive [PPV] and negative predictive value [NPV]) using a combination of two to three parameters are presented. Parameters included were overall score, relative weight loss, general condition score, spontaneous behaviour score, and daily analgesic requirements as assessed by the observer, each on the second post-operative day

					Pearson’s Chi-square test			
Parameters (with cut-off values) on post-operative day 2	Sens (%)	Spec (%)	PPV (%)	NPV (%)	χ^2^ value	df	*P-*value	Phi
Overall score (≥ 14) + DARAO (≥ 0.5)	66.7	100	100	98.7	105.299	1	< 0.001	0.811
Relative weight loss (≥ 12.3%) + DARAO (≥ 0.5)	83.3	97.4	55.6	99.3	71.373	1	< 0.001	0.666
Relative weight loss (≥ 12.3%) + general condition score (≥ 1.5)	71.4	95.5	41.7	98.7	43.721	1	< 0.001	0.519
DARAO (≥ 0.5) + general condition score (≥ 1.5)	66.7	96.1	40	98.7	39.101	1	< 0.001	0.493
Relative weight loss (≥ 12.3%) + DARAO (≥ 0.5) + general condition score (≥ 1.5)	66.7	98.1	57.1	98.7	58.199	1	< 0.001	0.601

DARAO: daily analgesic requirements as assessed by the observer; df: degrees of freedom; NPV: negative predictive value; Phi: symmetric measure Phi; PPV: positive predictive value; Sens: sensitivity; Spec: specificity.

### Complications and their consequences

During the experiments, the following three complications were observed which were not listed in the score-sheet: (i) Horner’s syndrome (after the second surgery in 57% [49/86] of the rats; 28 of the sham animals and 21 of the BDL group) (Figure 7[a]); (i) peritoneal rupture with herniation (four BDL rats and one sham animal after the first surgery) (Figure 7[b]); and (iii) wound dehiscence (in 20 rats [BDL: n = 4; sham: n = 16]). Although the severity of the complications differed among the rats, the animals did not appear to be affected by any of them. For Horner’s syndrome, no treatment was needed, and partial or full recovery was observed in 17 (17/28; 61%) animals in the sham group and eleven (11/21; 52%) animals in the BDL group over time. Peritoneal rupture repair, however, was performed routinely during the second surgery and wound dehiscence required repair in short anaesthesia in case the subcutaneous suture had been exposed.

### Data availability

The datasets generated and analysed during the current study are available from the corresponding author on request.

## Discussion

Using all the data documented during the experiment, the severity for the animals caused by different influences (such as the surgery and cirrhosis as a pathologic condition) and their interactions were analysed. The detailed assessment of the individual parameters and their combinations could improve the predictive value of the original score-sheet.

### Severity assessment of the procedures

In this experiment, the different surgeries (BDL, sham or vascular), cirrhosis as pathological condition, or an interaction of both factors could have caused animal severity. The short-term severity was higher after the BDL surgery than after the sham surgery (higher mortality and higher need for analgesics). Mean severity values were highest on the second post-operative day. This time-point is consistent with the maximum of acute cholestatic liver damage following BDL (peak of ALT and biliary infarction) described in the literature (Georgiev *et al*. 2008).

A higher number of animals died or had to be euthanased after the first compared to after the second surgery. Most of them showed myocardial infarction, a complication of an acute event (Uhlig *et al*. 2021), not being detectable by the score-sheet. Even though no animal died after the second surgery (vascular damage), the animals fared worse than after the first surgery (abdominal surgery: BDL or sham), as evidenced by higher overall score values and, especially in the BDL group, a prolonged demand for analgesics. This is the opposite of what the location and complexity of the surgeries would have suggested and indicates that the rats were more sensitive to a second surgical procedure despite a four-week interval with good recovery. For the design of longitudinal studies this observation has important implications, as repeated measurements within one animal may reduce the number of animals needed but increases the risk of complications and the severity level.

Except immediately post-operatively, cirrhosis *per se* (in the eight weeks after BDL induction), as an underlying co-morbidity, had no overall effect on severity (no significant group difference in the overall score or absolute BW). Nevertheless, the prolonged and increased need for analgesics after the second surgery in the BDL group implies that BDL rats might be more susceptible to the effects of a second surgery.

### How to evaluate score parameters?

A major issue with the assessment of score parameters is the right method: when parameters are assessed by their ability to discriminate between two conditions that are most likely associated with different levels of severity (e.g. sick and healthy), there is a risk that procedure- or disease-specific parameters will be incorrectly evaluated as good parameters for severity. For example, jaundice correlates well with the induction of liver cirrhosis, but not with the level of severity experienced by an individual animal and is thus not valuable in evaluating the severity. Therefore, we focused instead on the predictive power of a score parameter for survival, using ROC analyses.

However, there are also ethical aspects to consider when evaluating score parameters: the highest goals of ‘early detection and elimination of suffering’ and at the same time ‘not killing animals unnecessarily’ sound excellent but are by no means unambiguous. The second raises, for example, the question of what is unnecessary in this context. It might be a ‘price worth paying’ to kill some animals that would have survived the experiment if it avoided the suffering and death of moribund animals that could have been killed earlier as a result. In addition, one must consider that an animal that survives the experiment, but that cannot be distinguished from those that will die in the course, is probably in very poor condition at times and thus may suffer disproportionately. However, it is not the aim of this study to resolve these ethical issues, nor is it possible to do so. We will thus limit the subsequent assessment of the score parameters to the interpretation of the statistical aspects.

### Assessing the original score-sheet

Despite an optimised cut-off, the overall score did not satisfy in predicting survival: a PPV of 50% would mean that 50% of the euthanased animals would be euthanased in vain. These numbers were even worse for individual parameters (PPV < 50%; Figure 6). Furthermore, the following two individual subcategories of the used score-sheet did not prove helpful in predicting survival at all: (i) procedure specific variables (the parameters listed did not occur); and (ii) spontaneous behaviour (no benefit in predicting survival in the ROC analysis despite optimised cut-off value; Figure 6). Retrospectively, the procedure-specific parameters listed in the score-sheet were probably not appropriate for our study design. Therefore, other parameters, linked to wound healing (including wound dehiscence), peritoneal rupture with herniation requiring therapy or the parameter of Body Condition Score which represents the animals’ constitution regardless of BW effecting circumstances such as tumours or enlarged organs should be tested for their usefulness in future studies. For better comparability with other research groups and to increase consistent decision-making within a research group, a more precise description of the score parameters is recommended than was originally used in this study. This was corrected in this study by subsequently adding footnotes to Table 1.

### Alternative parameters

A good overview of alternative, non-invasive tests for pain in rodents is provided by Tappe-Theodor and Kuner (2014). However, the disadvantage of most of these methods is that the animals have to be kept individually which, in turn, might also have a negative effect on their general condition especially for long-term experiments. In addition, some of these tests are not suitable for repeated use, induce an impact on the animal, showed inconsistent results in previous studies, or are insufficiently validated. A recently more frequently used tool is the observation of animals through video monitoring, either in their home cage or in test situations such as the open field, which seems suitable for various models (Ernst *et al*. 2020; Zieglowski *et al*. 2020, 2021). Nevertheless, an ideal test does not yet exist and further studies investigating the quantification of severity are urgently needed.

### Most valuable score parameters

Despite high examiner dependency, the number of daily needed pain medication (DARAO) on the second post-operative day proved to be the individual parameter with the best predictive power for survival (highest AUC; Figure 6), followed closely by relative BW loss. However, it should be noted that the examiner’s assessment of required pain medication, as opposed to the relative change in BW, can only be judged within this experimental setting. In other laboratories, with other observers, a completely different picture may emerge. The overall score, general condition score and spontaneous behaviour score (predictive power descending in that order) also seemed helpful in predicting survival given AUC and *P*-value. However, PPV values for all individual parameters were poor (maximum 50%).

### Cut-off values and combining parameters

A high PPV, along with high specificity, is crucial, though, in order to euthanase as few animals incorrectly as possible. A combination of score parameters (with the new cut-off values) yielded better results in this respect (Table 4). Altogether, the best way to evaluate the rats in this study would have been through a combination of the DARAO (threshold ≥ 0.5) and the overall score (threshold ≥ 14 score points) on the second post-operative day. As a result, no animal would have been incorrectly euthanased (false positive) and only two (instead of originally four) rats would have died due to not being detected (false negative). Nevertheless, the calculated cut-off values cannot be applied to the model in general; this would require further validation in larger study cohorts. However, the results indicate that a combination of several parameters (using the ‘believe the negative’ rule) is superior to individual parameters for euthanasia decision-making. This questions the performance of the frequently accepted humane end-point ‘BW loss’ as a single parameter (Liedtke *et al*. 2013). Similar considerations, of combining parameters rather than relying on a single parameter, have been advocated by various authors (Morton & Griffiths 1985; Baumans 2005; Tang *et al*. 2020).

### Limitation

As with most scores, a limitation of the used score-sheet was that, with exception of BW, subjective, observer-dependent parameters were used. Nevertheless, consistent with our findings, Hawkins and Morton (2011) underline the importance of using subjective, experienced, human judgment as a complement to an objective evaluation. In addition, the retrospective nature of the study limits its power. Due to statistically rare negative events (n = 7 post-operative death) and relatively small sample size (n = 87 rats), it was not reasonable to divide the rats into a first cohort for the development of a new score-sheet and a second for its validation. Therefore, a validation cohort is missing in this study, which means that the validation of the new cut-off values is still pending and needs to be carried out in subsequent studies. Even though BW is an objective parameter, the interpretation of BW changes have to be carried out with caution. For example, a steeper slope of BW gain after the first surgery may have simple physiological reasons: the animals were older after the second surgery and therefore may have gained BW more slowly. Additionally, higher BW gain after the second surgery in the BDL group could be due to a higher liver weight.

### Animal welfare implications

It is essential to consider the well-being of animals when using them in experimental studies. The ethical discussion about when the killing of an animal is necessary and required can be a very difficult decision for a researcher who wants, on the one hand, to advance the experiment while, on the other, protect the animals from severe suffering. Many aspects have to be taken into account. Score-sheets can therefore be an important tool in the decision-making process. It is also advisable for difficult cases to be decided in a team that explicitly includes an animal welfare advocate. This can ensure acting in the animal’s best interest.

The present work has clearly demonstrated that the score-sheet used was far from ideal for use as a humane endpoint in rat experiments. Nonetheless, evidence-based evaluation could significantly improve the prediction by adding another parameter to the overall score: DARAO. DARAO was based on the overall assessment of experienced experimenters who took time to study the animals. Incorporating such factors of experience and human judgement into traditional score-sheets (in this case by adding the assessment of analgesic need) might help to refine experiments, minimise unnecessary suffering and euthanasia, and thereby reduce the number of animals needed (3Rs principle) (Russell & Burch 1959). It is possible that factors of human experience and judgement have been underestimated so far. However, when using them, one must bear in mind that they are heavily biased variables that are highly dependent on the investigator. This study can only assess the investigator’s judgement in the setting presented.

Although the use of score-sheets (even without explicit subjective factors) is not entirely objective and far from perfect, it is the best available option to optimise animal welfare during experiments. In any case, one thing should be certain and common practice for all humans working with animals: when in doubt the decision must be made in the best interest of the animals.

Furthermore, the study also improves the understanding of the severity rats experience from consecutive surgeries and cirrhosis as a pathologic state.

## Supplementary material

For supplementary material accompanying this paper visit https://doi.org/10.1017/awf.2023.21. click here to view supplementary material
