# Supplementary material for: Evaluation of score parameters for severity assessment of surgery and liver cirrhosis in rats
Source: Anim Welf. 2023 Mar 10;32:e29. doi: 10.1017/awf.2023.21 (PMC10936376; doi:10.1017/awf.2023.21)
Supplement: Supplementary file 1 [file S0962728623000210sup001.pdf]

Supplemental file

S1.

Sample processing

Clinical chemistry

Serum alkaline phosphatase (AP), aspartate aminotransferase (AST), alanine aminotransferase (ALT), gamma-glutamyl transferase (GGT), lactate dehydrogenase (LDH), albumin, total bilirubin, creatinine, lactate and urea levels were measured using an automated analyser (System Chemistry Vitros 350, Ortho Clinical Chemistry Diagnostics, Johnson & Johnson Company, New Brunswick, NJ, USA) after 4 and after the experimental duration of 3, 7, 14, and 28 days.

Histological specimens

Liver tissues were fixed in 4% formaldehyde, dehydrated through an ascending ethanol gradient, embedded in paraffin, cut into 3-µm-thick sections and stained with haematoxylin and eosin (HE) or Sirius red as described previously (Roehl et al 2013).

Histological sections from the livers were examined via light microscopy using a LEICA DM 2500 microscope (Leica Camera AG, Wetzlar, Germany). Images were captured with a LEICA DFC 420C camera. All slides were photographed under bright field illumination at ×10 magnification. To ease collagen quantification, slides from the 28 days group stained with Sirius red were additionally imaged under polarisation contrast illumination at ×10 magnification to directly visualise collagen fibres. Lamp intensity, camera exposure and camera gain were kept constant. Moreover, attention was paid to orthogonal polarising filter placements to unify the dark background as much as possible.

Histological image analysis

Liver cirrhosis was classified by a blinded veterinary pathologist. Light microscopic sections of the three segments (right, middle and left lobes) of the liver of the animal in the 28-day group were examined. In total, six parameters (bile duct proliferation, fibrosis, vacuolar degeneration, cell infiltration, necrosis and haemorrhage) were evaluated and scored for each segment on a 4-point scale as follows: 0, no alteration from the physiological condition; 1, mild deviations; 2, moderate deviations; 3, severe deviations and 4, extremely severe deviations. Analysis and representative images for this classification are shown in the Supplementary Figure 1 (S1) and Supplementary Figure 2 (S2). For the standardised quantification of liver fibrosis, 10 non-overlapping fields of vision without any part of the liver capsule were randomly and separately selected from the right, left and medium lobes of the liver of each animal. The images were analysed using *Fiji* to quantify fibrosis (Schindelin et al 2012). Initially, the region of interest was defined. Fissures and perivascular regions were manually excluded as described previously (Street et al 2014). Subsequently, a background intensity threshold was set, and the collagen-containing tissue area was calculated. The degree of fibrosis was expressed as the ratio between the collagen-containing tissue area and total tissue area.

Statistical analysis

Fisher's exact test was used for group comparisons of the histological scores performed by a blinded veterinary pathologist.

S2.

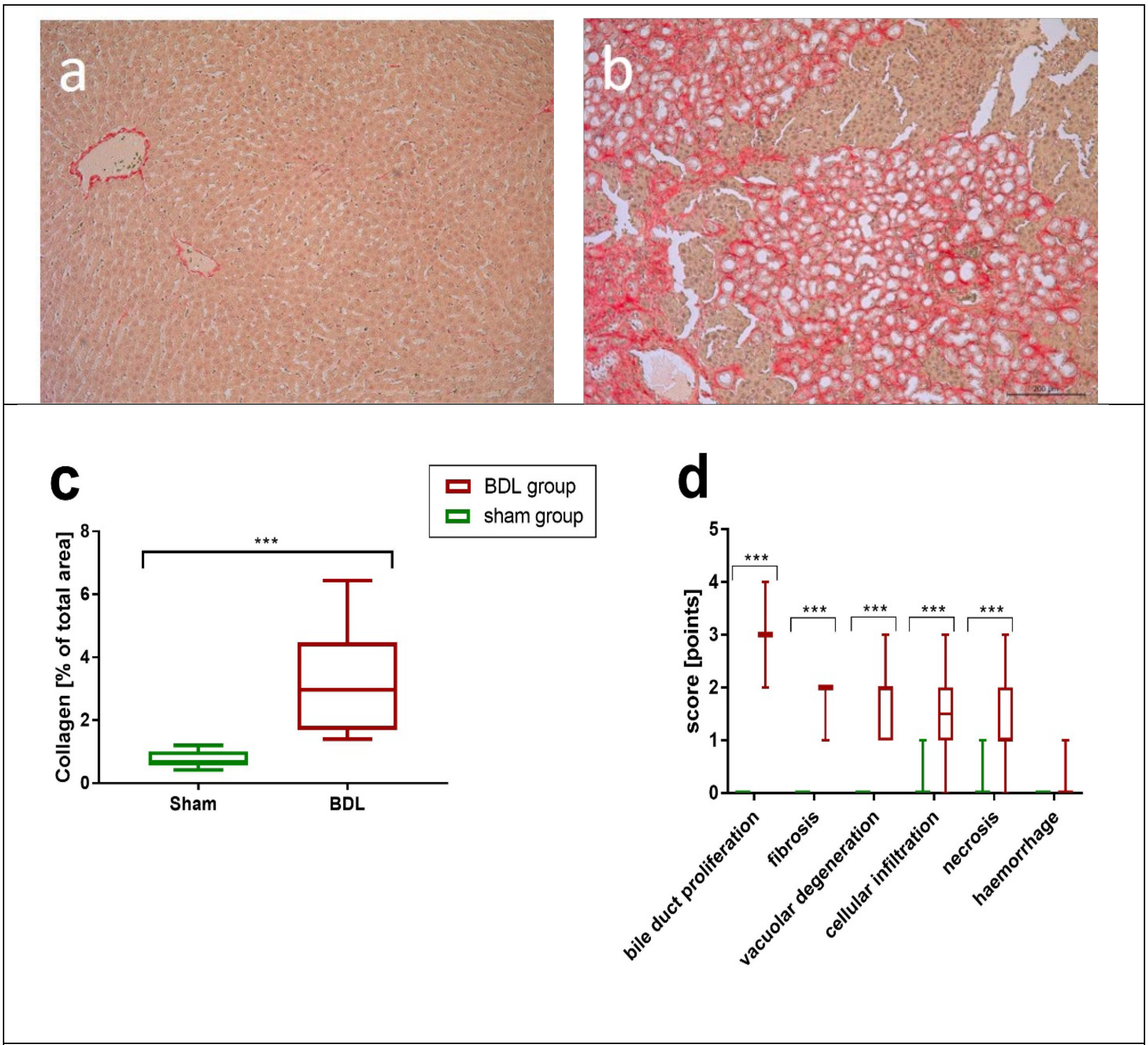

S2: Comparison of histological specimens of rat livers 8 weeks after bile duct ligation (BDL, red) or sham surgery (green). Representative Sirius red-stained specimens from (**a**) the sham and (**b**) BDL groups are shown at ×10 magnification under a light microscope. (**c**) Collagen content relative to the total area, as determined using automated and standardised analysis under polarised light, is shown separately for the two groups. (**d**) Severity of liver cirrhosis based on the scores for bile duct proliferation, fibrosis, vacuolar degeneration, cellular infiltration, necrosis and haemorrhage (0 = no alteration, 1 = mild, 2 = moderate, 3 = severe and 4 = extremely severe). Median, minimal and maximal values with interquartile ranges are presented. *** $P < 0.001$ between groups.

S3.

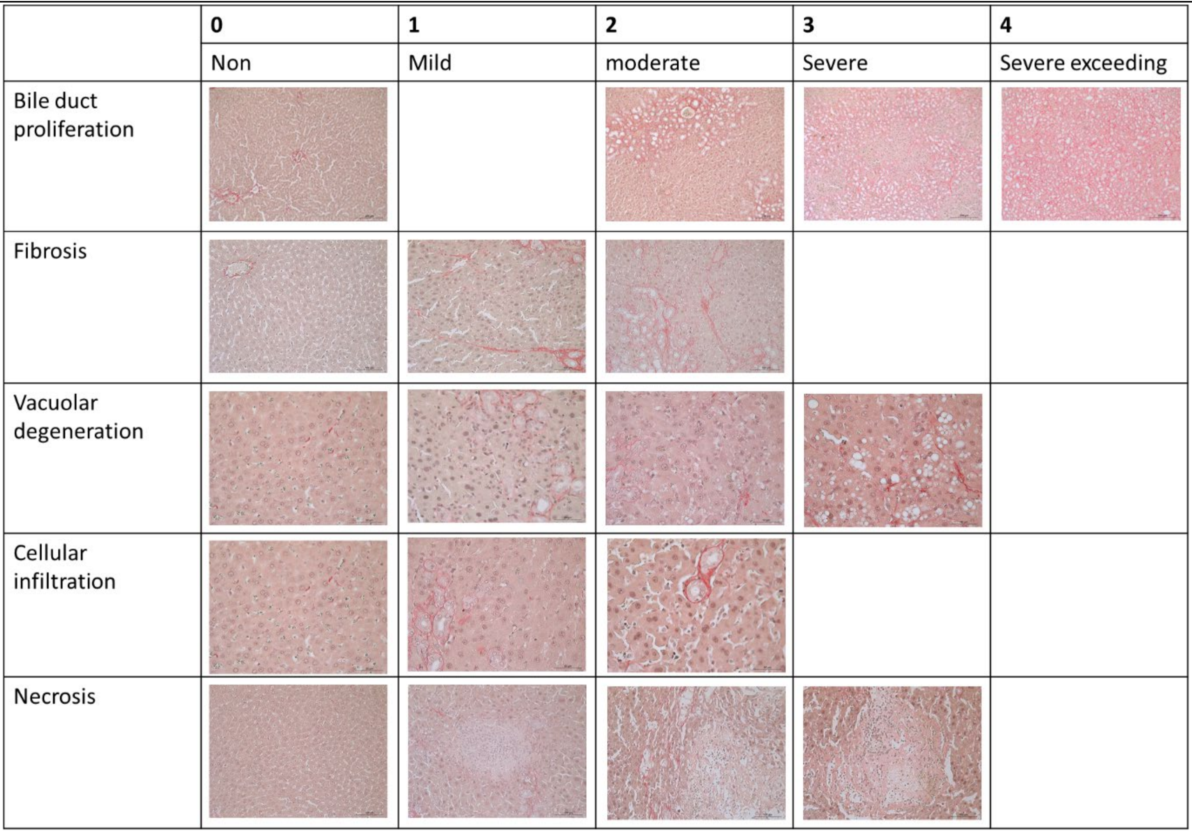

S3. Representative Sirius red-stained specimens of rats presenting the classification of liver cirrhosis (0, none; 1, mild; 2, moderate; 3, severe; 4, extremely severe) based on different parameters (bile duct proliferation, fibrosis, vacuolar degeneration and cellular infiltration, necrosis) using light microscopy. Free squares indicate that the parameters were not visible. Bile duct proliferation is presented at ×10 magnification, fibrosis and necrosis are shown at ×20 magnification and vacuolar degeneration and cellular infiltration are shown at ×40 magnification.

S4.

S4. Clinical chemistry (AP, AST, ALT, GGT, LDH, urea, creatinine, total bilirubin, albumin, and lactate): values were measured during the second surgery (4 weeks after BDL or sham surgery) and during the final surgery (3, 7, 14, and 28 days after the second surgery (balloon dilatation of the carotid artery)) in the sham and bile duct ligation (BDL) groups. Values are presented as mean ± standard deviation; p-values were calculated using analysis of variance with the group and number of surgeries as covariates.

Blood values		Second surgery			Final surgery after 3d			Final surgery after 7d			Final surgery after 14d			Final surgery after 28d		
	P_{group}	**Sham n = 40**	**BDL n = 43**	$P_{surgery}$	**Sham**	**BDL**	$P_{surgery}$	**Sham**	**BDL**	$P_{surgery}$	**Sham**	**BDL**	$P_{surgery}$	**Sham**	**BDL**	$P_{surgery}$
AP [U/L]	<0.001	249 ± 66	326 ± 119	<0.001	174 ± 56	426 ± 238	0.007	192 ± 46	349 ± 268	0.085	216 ± 47	273 ± 55	0.029	241 ± 116	332 (± 67)	0.047
AST [U/L]	<0.001	82 ± 34	389 ± 328	<0.001	77 ± 20	707 ± 362	<0.001	82 ± 40	320 ± 195	<0.001	76 ± 15	301 ± 98	<0.001	82 ± 40	349 (± 130)	<0.001
ALT [U/L]	<0.001	54 ± 12	102 ± 117	0.012	38 ± 5	123 ± 56	<0.001	47 ± 7	54 ± 15	0.186	44 ± 7	62 ± 17	0.005	55 ± 21	80 (± 32)	0.057
GGT [U/L]	0.062	6 ± 4	40 ± 44	0.008	0 ± 0	187 ± 309	0.445	5 ± 0	39 ± 25	0.254	5 ± 0	39 ± 22	0.070	28 ± 72	51 (± 30)	0.374
LDH [U/L]	0.619	467 ± 597	707 ± 913	0.179	834 ± 967	521 ± 537	0.097	582 ± 427	425 ± 456	0.479	807 ± 731	784 ± 602	0.942	588 ± 581	474 (± 437)	0.626
Urea [mmol/L]	0. 008	7 ± 0.6	14 ± 19	0.015	8 ± 2	10 ± 1	0.341	6 ± 0.5	8 ± 3	0.042	6.6 ± 0.7	7.8 ± 0.6	<0.001	10 ± 7	9 (± 1)	0.660
Creatinine [µmol/L]	<0.001	32 ± 8	44 ± 28	0.013	45 ± 13	52 ± 20	<0.001	36 ± 10	43 ± 6	0.097	31 ± 7.5	38 ± 5	0.040	32 ± 12	44 (± 7)	0.010
T. bilirub. [µmol/L]	<0.001	13 ± 33	147 ± 121	<0.001	3 ± 1	110 ± 40	0.030	4 ± 2	135 ± 29	<0.001	4.3 ± 0.8	149 ± 52	<0.001	7 ± 7	152 (± 23)	<0.001
Albumin [g/L]	<0.001	27 ± 2	26 ± 4	0.207	27 ± 2	25 ± 2	0.072	27 ± 2	23 ± 3	0.004	26 ± 3	25 ± 2	0.269	28 ± 2	25 ± 2	0.016
Lactate [mmol/L]	0.705	2 ± 0.7	2 ± 0.9	0.690	3 ± 0.6	2 ± 1	0.614	2 ± 0.6	3 ± 1	0.037	1.7 ± 0.3	1.5 ± 0.7	0.614	1.2 ± 0.6	1.4 ± 0.4	0.562

Abbreviations. AP, alkaline phosphatase; AST, aspartate aminotransferase; ALT, alanine aminotransferase; GGT, gamma-glutamyl transferase; LDH, lactate dehydrogenase; T. bilirub., total bilirubin

S5.

S5. Absolut liver weight (including bile), bile amount, ascites amount, and absolute liver weight (excluding bile) measured at the end of the final surgery (3, 7, 14, and 28 days after balloon dilatation of the carotid artery) in the sham and bile duct ligation (BDL) groups. Values are presented as mean ± standard deviation; P-values were calculated using a *t*-test.

	Final surgery											
Days after 2nd surgery	3			7			14			28		
**Pameter**	**Sham**	**BDL**	**P**	**Sham**	**BDL**	**P**	**Sham**	**BDL**	**P**	**Sham**	**BDL**	**P**
Absolute liver weight (including bile) [g]	13.7 ± 2.0	31.0 ± 6.7	<0.001	15.1 ± 1.2	36.6 ± 8.3	<0.001	15.4 ± 1.8	42.2 ± 7.2	<0.001	16.0 ± 1.8	53.9 ± 5.9	<0.001
Bile amount [mL]	0 ± 0	3.2 ± 1.7	<0.001	0 ± 0	6.2 ± 5.8	0.020	0 ± 0	7.1 ± 5.2	0.002	0 ± 0	9.1 ± 2.9	<0.001
Ascites amount [mL]	0 ± 0	2.7 ± 2.9	0.030	0 ± 0	11.0 ± 16.3	0.098	0 ± 0	5.9 ± 4.8	0.004	0 ± 0	9.9 ± 16.0	0.081
Absolute liver weight (excluding bile) [g]	13.7 ± 2.0	27.8 ± 5.9	<0.001	15.3 ± 1.0	29.4 ± 4.3	<0.001	15.5 ± 2.1	35.0 ± 5.8	<0.001	16.4 ± 2.0	44.9 ± 5.7	<0.001

S6.

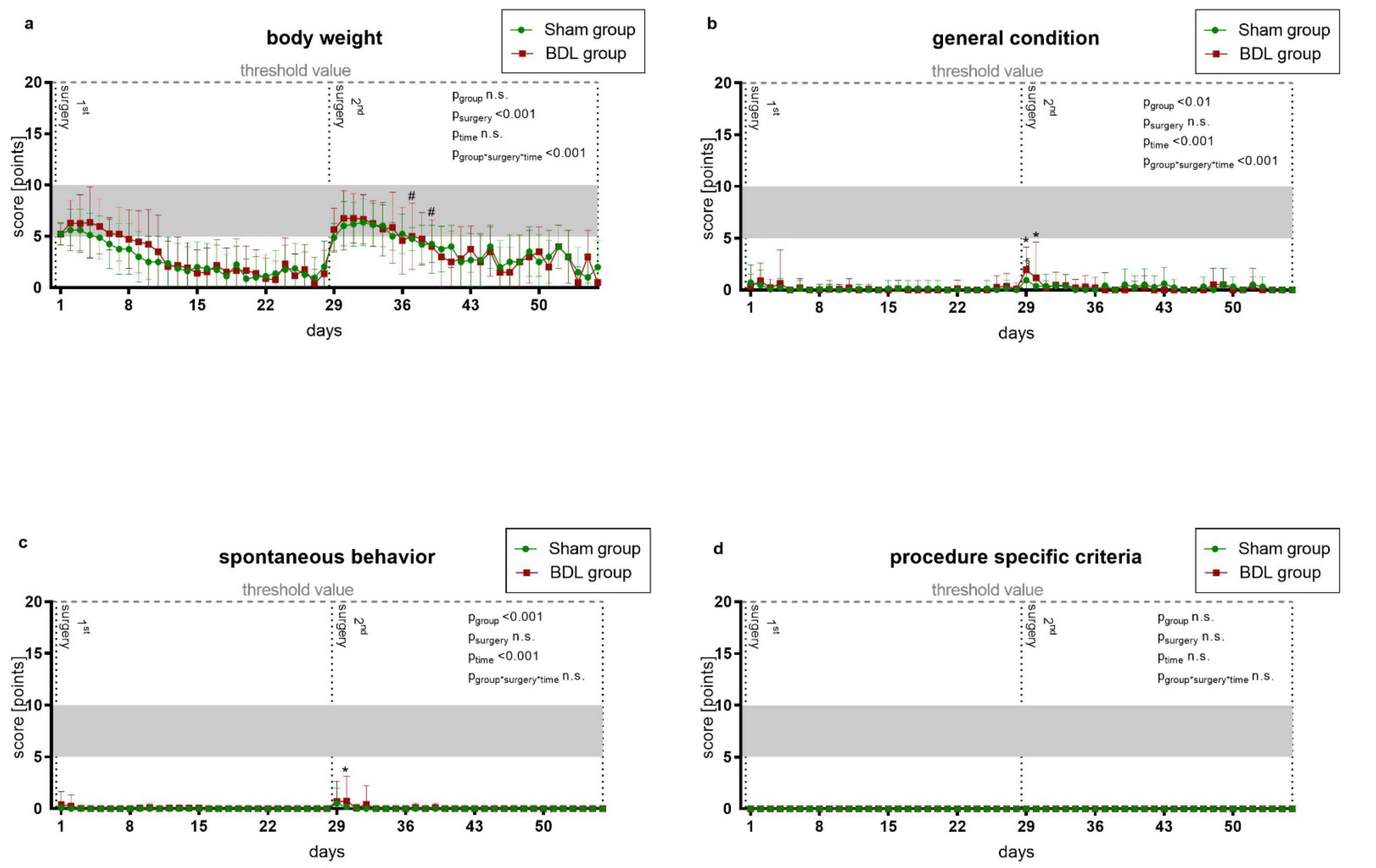

S6. Time course of changes in the scores for (**a**) body weight, (**b**) general condition, (**c**) spontaneous behaviour, and (**d**) procedure specific criteria for the bile duct ligation (BDL, red) and sham groups (green). The single score categories were analyzed using a generalized estimating equation for group, number of surgeries and days after surgery. *t*-tests with adjustment for multiple comparison were used for post hoc comparisons. Values are presented as mean ± standard deviation; P-values were derived from generalised estimated equations. *P < 0.05, post hoc analysis between groups; #P < 0.05, between surgeries within the sham group; ns - no significance. Mild severity is depicted by the grey-shaded area.
